# Performance Assessment of Three Similar Dental Restorative Composite Materials via Raman Spectroscopy Supported by Complementary Methods Such as Hardness and Density Measurements

**DOI:** 10.3390/polym16040466

**Published:** 2024-02-07

**Authors:** Stefan-Marian Iordache, Ana-Maria Iordache, Dina Ilinca Gatin, Cristiana Eugenia Ana Grigorescu, Roxana Romanita Ilici, Catalin-Romeo Luculescu, Eduard Gatin

**Affiliations:** 1 Optospintronics Department, National Institute for Research and Development in Optoelectronics—INOE 2000, 077125 Magurele, Romania; stefan.iordache@inoe.ro (S.-M.I.); ana.iordache@inoe.ro (A.-M.I.); krisis812@yahoo.co.uk (C.E.A.G.); 2 Faculty of Dentistry, University of Medicine and Pharmacy “Carol Davila”, 020021 Bucharest, Romania; dinailinca@yahoo.com (D.I.G.); roxana.ilici@umfcd.ro (R.R.I.); 3 National Institute for Laser, Plasma and Radiation Physics, CETAL, 077125 Magurele, Romania; 4 Faculty of Medicine, University of Medicine and Pharmacy “Carol Davila”, 020021 Bucharest, Romania; 5 Faculty of Physics, University of Bucharest, 077125 Magurele, Romania

**Keywords:** dental materials, microhardness, density of dental composites, Raman spectroscopy, cavity treatment

## Abstract

(1) Background: A widespread problem in oral health is cavities produced by cariogenic bacteria that consume fermentable carbohydrates and lower pH to 5.5–6.5, thus extracting Ca^2+^ and phosphate ions (PO_4_^3−^) from teeth. Dental restorative materials based on polymers are used to fill the gaps in damaged teeth, but their properties are different from those of dental enamel. Therefore, a question is raised about the similarity between dental composites and natural teeth in terms of density and hardness. (2) Methods: We have used Raman spectroscopy and density and microhardness measurements to compare physical characteristics of several restorative dental composites at different polymerization intervals. (3) Results: XRVHerculite^®^, Optishade^®^, and VertiseFlow^®^ showed the very different characteristics of the physical properties following four polymerization intervals. Of the three composites, OptiShade showed the highest polymerization rate. (4) Conclusions: Only fully polymerized composites can be used in teeth restoring, because incomplete polymerization would result in cracks, pitting, and lead finally to failure.

## 1. Introduction

Cavities are an omnipresent problem in oral health, developed by children (60–90%) and adults alike (90–100%) [1]. Tooth decay is produced by cariogenic bacteria that consume fermentable carbohydrates and lower the pH to 5.5–6.5, thus extracting Ca^2+^ and phosphate (PO_4_^3−^) ions from teeth [1,2]. The reversible process, deposition of Ca^2+^ and PO_4_^3−^ ions from saliva tries to compensate the loss and reconstitute hydroxyapatite (Ca_10_(PO_4_)_6_(OH)_2_) in enamel. If the process is maintained in equilibrium, the cavities do not form; however, an imbalance is present most of the time, and small caries initiate advanced demineralization and lead to tooth decay.

To arrest the demineralization process, clinical restorative polymer materials (e.g., resin composites, adhesives, and dental primers) are used to replace missing enamel/dentin tissue. However, all replacement polymers contain either bisphenol A glycidyl dimetacrylate (bisGMA) or triethylene glucol dimethacrylate (TEGMA), which promote mineral imbalance and sustain biofilm development on the restored tooth [1]. Moreover, the recurrence rate for this type of cavities is around 60% [1]. In a systematic review analyzing the materials used for the treatment of carious tissue, the American Dental Association recognized the superiority of amalgam in terms of durability, longevity, and affordability [3], but the use of mercury has raised concerns about health security and environmental harms. Thus, using dental composites for the treatment of damaged teeth was a better alternative than amalgam. To achieve the best results in terms of durability, the physical properties of the dental composites should be closely matched to those of the enamel.

There are a series of tests that can be conducted on the dental restorative materials: stress–strain ratio, elastic modulus, Poisson ratio, flexural strength, resistance to fatigue, and hardness [4] to name a few. In terms of mechanical properties, Chun and Lee [5] tested six dental restorative materials (amalgam, dental ceramic, gold alloy, dental resin, zirconia, and titanium alloy). Apart from dental ceramic, all the other materials exhibited maximum stress and strain values higher than natural enamel and dentin. Zirconia was particularly interesting because its hardness value was 4.5 over that of enamel, which implies poor biocompatibility. Since dental restorative materials have to sustain prolonged biting wear (vertical and side compression forces) they have to be compatible with the surrounding natural teeth components (from the Vickers’ hardness values, dental resin and titanium alloy were best matched with dentin and enamel, respectively). Chung et al. [6] has proposed the measurement of Poisson’s ratio for dental composite materials to determine their mechanical characteristics including biaxial flexural strength and indentation modulus. A strong mismatch between the elastic values of the restorative materials and natural tooth will cause micro-fractures which could lead to composite failure.

This is seen in the case of resin composites that more commonly undergo surface degradation and margin/bulk fractures [7]. The authors conducted a study on four resin composites assessing the fracture toughness, Vickers hardness, and color change, among others, in dry and wet environments. Their conclusion was that moisture had a direct influence on the hardness and fracture propagation of the resin materials due to the polymerization shrinkage stress (materials that contain a polymerization modulator are less capable of leaching their un-polymerized monomers).

Yadav et al. used statistical analysis and different decision-making processes to rank dental restorative composite materials [8,9] or tribological behavior [10] to assess the compatibility between hybrid dental restorative composite materials and natural teeth. Tribological characterization has also been employed by Amanda Carvalho et al. [11] to evaluate the shear stress in restorations and to compare the wear behavior of these materials with patients’ teeth. Other authors [12,13,14] evaluated the wear behavior by using a dual-axis chewing simulator and/or an ACTA wear machine in order to establish a relationship between the physical parameters and wear of dental composites [15].

Besides the mechanical and esthetical properties, dental materials should also exhibit antimicrobial activity. An excellent review [16] showed that the preferred materials are the positively charged nanoparticles which can interact with the negatively charged membrane of the bacteria, penetrating the cellular wall and destroying the cell. The nanoparticles do not interfere with all the other properties (flexural strength, modulus of elasticity, and hardness, self-curing, radiopaque, etc.), but complete this list with their sustainable bacterial resistance. A good example is the use of strontium phosphate glass microfiller, which can inhibit the microbial growth of *S. mutans* while maintaining the mechanical properties [17]. A major drawback is, however, the release of Ca, P, and Sr in the oral environment (its release increases with the increase in the strontium phosphate concentration in the filler).

The physical characteristics of the restorative materials change over time and after harmful repetitive actions such as aesthetic bleaching and intense ultrasound cleaning [18,19]. These actions could result in damages to both natural teeth and restorative materials. Thus, assessing the compatibility between filler and natural teeth is important during yearly checkups. The main drawback of these methods is the requirement of specialized instruments. Moreover, they are sensitive to the variation in porosity and density of the dental filling materials.

Our group has been investigating the compatibility between several commercially available dental restorative materials and natural enamel [20,21,22,23,24,25]. In this paper, we aim to evaluate the physicochemical properties of three commercially available and widely used dental materials and rank them according to those properties. In our investigations, we used simple and affordable instruments and correlated the chemico-structural characteristics with the behavior of the composite. We selected three dental restoration materials produced by the same company (Kerr Corp., Orange, CA, USA) to maintain a similar composition among the samples. The manufacturer aimed to improve (or at least to maintain) the mechanical properties of the old dental restoration materials (e.g., Herculite), but in the meantime was interested in adding esthetic features and easy handling for dental cavity application (filling)—according to the release date of the products in Table 1. We focused on highlighting the Raman spectroscopy results because it corroborated the results from the microhardness and density measurements and provided further insights into the photopolymerization process, allowing us to calculate the degree of conversion of the monomer and to rank the materials in terms of stability.

## 2. Materials and Methods

### 2.1. Materials

We selected three of the most widely used dental restorative materials in Romania and ranked them in terms of microhardness, density, and degree of polymerization (curing). Thus, XRV Herculite^®^, VertiseFlow^®^, and Optishade^®^ composite fillers were purchased from Kerr (Kerr Corp., Orange, CA, USA) [26] and each sample was photopolymerized for four different time intervals (5 s, 10 s, 15 s, and 20 s) before testing.

According to the datasheet, XRV Herculite^®^ contains 41% methacrylate ester monomers and 59% inert mineral fillers (average particle size of 0.6 microns). VertiseFlow^®^ is composed of about 30% methacrylate monomers and less than 10% ytterbium trifluoride. OptiShade^®^ contains 80% fillers and 20% monomers, activators, and stabilizers. The complete composition, based on the datasheet and references [27,28,29], is presented in Table 1.

Curing was performed with a Denjoy DY400-4 LED lamp (λ = 420–480 nm, from Kerr Corp., Orange, CA, USA), which delivers a power of 720 mW and has a light intensity at the irradiation plane in the 1500–2000 mW/cm^2^ range.

### 2.2. Methods

Density measurements were performed at 18.6 °C by the pycnometer method (pycnometer from Paul Marienfeld Gmbh, Lauda-Königshofen, Germany) using a six-digit microbalance. For this measurement, a plastic mold of 5 mm diameter and 2 mm thickness was used for the preparation of the disc samples of each restorative material; the disc samples were cured with the LED lamp at four curing durations (5 s, 10 s, 15 s, and 20 s). Our reason behind producing samples of this geometry is that clinical gross dental cavities are about 5 mm (width) and 2 mm (depth), hence the geometrical dimensions (5 × 2 mm) for our mold. For larger cavities, the restoration material must be added in layers to overlap with the correspondent irradiation time exposure on photo polymerization lamp in order to have a final good quality dental restoration work.

Microhardness was tested by means of the Vickers method and was measured on the back side of the samples as the face surface was deformed due to shrinkage (the surface at the sample/air interface released the volatile compounds faster than the bottom surface and the samples became concave). The tester was equipped with a square based diamond pyramid as indenter; we set the value of the load to 25 g and the time duration of 10 s. The sample was held firmly in position and the indentation performed using the set parameters. The equipment used in our investigation was a microhardness tester Model FM-700 and Serial number XM0190 (Future-Tech Corp, Kawasaki, Japan). The obtained results are given by unit of hardness that are known as the Vickers pyramid number (HV). The Vickers test is often easier to use than other hardness tests since the required calculations are independent of the size of the indenter, and the indenter can be used for all materials irrespective of hardness.

The Raman spectra were obtained with a research-grade dispersive micro-Raman spectrometer (NRS-7200, from JASCO Corp, Tokyo, Japan) using a 531.94 nm laser beam as a probing source. The laser light was focused on the sample surface by means of a long working distance 10× magnification objective lens with a numerical aperture (NA) = 0.25 (Olympus, Tokyo, Japan) to a spot of about 20 microns. The nominal power of the laser was set to 5.6–5.7 mW and spectra were collected in a backscattering geometry. The spectra were nonlinearly calibrated with PP bands. In some cases, a fluorescence correction was applied using JASCO Spectra Analysis 2.10 software using a circle type with 3–4 intervals and radii of 100–800 points. All spectra were analyzed in the OriginLab Origin Pro 9 software (version 9.6, OriginLab Corp, Northampton, MA, USA).

## 3. Results and Discussion

### 3.1. Density Measurements

Density measurements took into consideration the mass of the sample and the curing time and the results were adjusted for water density correction. Table 2 shows the results of the measurements. XRV Herculite had the highest density with a 20 s curing time. This value is similar to that of human enamel (2.84–3.00 g/cm^3^, according to [30]). The composition of the material—Herculite was composed of up to 59% mineral fillers—is responsible for this value. OptiShade, which had 80% nanofillers dispersed inside the monomeric matrix, has the lowest values for density. Even at 20 s curing time, the density barely approaches that of liquid water.

For the third composite, VertiseFlow, its behavior was curious: it reached the maximum density at 15 s of curing, while at 20 s the density was lowest. Ytterbium triflouride (YbF_3_) could be responsible for this anomaly, but the exact mechanism is not clear. Initially, YbF_3_ has been used as radiopacifying agent in composite resin restorative materials, but the additional benefits in terms of the lower setting time and fast hardening process have led to its widespread use in root-sealing cements. An excellent work on the intricate properties of this additive in dental restorative materials is given by John W. Nicholson [31]. Here, the author noted that the hardness of the surface of the restorative materials containing YbF_3_ and BaSO_4_ decreases at higher percentage.

This could explain the density measurements in VertiseFlow. YbF_3_ is a Lewis acid that releases fluoride when incorporated in polymer-based materials and leads to the formation of fluorinated compounds. The reaction is triggered by laser irradiation which causes local heating and accelerates the release of fluoride ions. In turn, fluorinated compounds typically have a low density [32]. For VertiseFlow, it is recommended that the curing time is limited to 15 s.

An important observation is that only for Herculite the density of this materials increases with the increase in the curing interval. For the OptiShade and VertiseFlow, the density does not vary in a homogeneous manner. This is due to the composition of the two composites: VertiseFlow has YbF_3_ as radiopacifier material and OptiShade has 80% nano-sized fillers finely dispersed in a monomer matrix, which becomes lightweight upon photopolymerization.

### 3.2. Microhardness Test

The Vickers hardness test produced hardness numbers (Vickers pyramid number—HV) that indicate the load over the indentation surface area. An important relation established by Equation (1) occurred between HV and the other materials’ constants, such as the E and YS:

(1) $$HV=\frac{2}{3}YS\;\{2+\ln\left[\frac{E}{3YStan\alpha }\right]\}$$

where,

- HV is hardness;
- E is the elastic modulus;
- YS is the yield strength;
- A is the semi-angle of the conical indenter.

According to the experiments and finite element analysis (FEA), Equation (1) is satisfied for E/Y tan α ˂ ~30; in case of E/Y tan α ˃ ~30, the relation is simple as H ˃ 3YS known as Tabor’s relation) or H~3YS (in this case, because our materials are polymers). HV behavior can be easily interpreted by the YS in the case of polymers [33].

**Figure 1:**
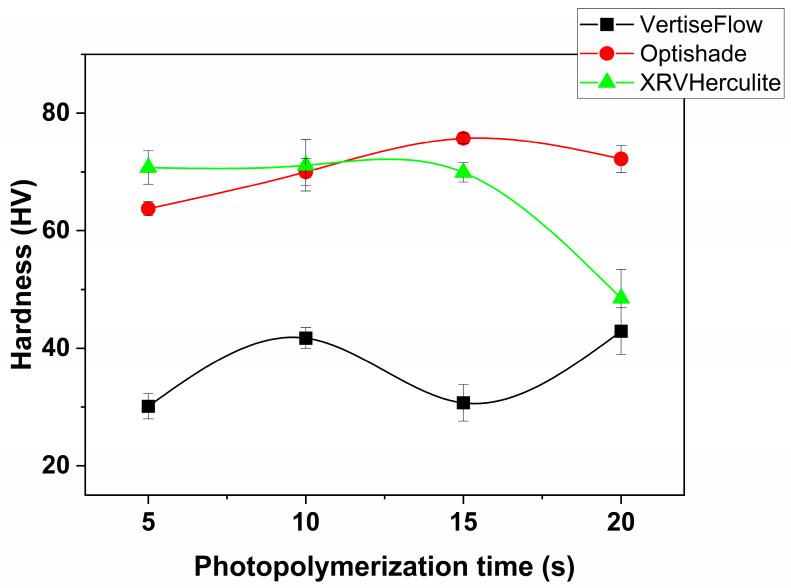
Vickers hardness results of the three dental materials after four polymerization intervals. Herculite and OptiShade reach the maximum hardness after 10 s and 15 s of polymerization, respectively, while VertiseFlow has an unusual behavior: the hardness value drops significantly at 15 s of curing compared to the neighboring values. This abnormal behavior could be due to the presence of YbF_3_, which reacts with the cured methacrylate polymer chains, breaking their bonds and weakening the structure.

Microhardness was measured on the back side of the samples as the face surface was deformed due to shrinkage. The results are presented in Figure 1. The highest HVs were obtained for OptiShade (the highest number was 75.7 HV for 15 s curing time). Compared with the HV for natural dental enamel, which is 273–374, OptiShade has a HV that is quite low. Herculite showed a decrease in hardness with the increase in curing time. Thus, its lowest HV was obtained after a 20 s curing time (48.48 HV) and its highest was for 10 s curing time (71.125 HV). This is unusual since Herculite comes the closest to natural enamel density. The lower hardness could be due to a lower concentration of fillers (only 59% compared to OptiShade, which has 80%) or to the size of the fillers (Herculite has micro-fillers, while OptiShade contains nano-sized fillers). Another relation was found between the monomer concentration and the HV, and is further sustained by the results for VertiseFlow. For the VertiseFlow composite, the HVs were the lowest of the set. This corresponds to a composition of 30% methacrylate. Moreover, these results come to support the density measurements where VertiseFlow showed an abnormal behavior for the 20 s curing time. We hypothesize that the YbF_3_ released during curing reacts with the cured methacrylate polymer chains, breaking their bonds, and weakening the structure (this justifies the low YS and low HV according to Equation (1) and the anomaly in density).

Corroborating these results with the density measurements we can conclude that OptiShade composite is lightweight and resistant, which makes it a very good material for dental enamel restorations. The results of the two measurements imply that the concentration of methacrylate polymers and the size of the fillers induce the level of hardness (the higher the concentration of fillers, the higher the HV) and the density (cross-polymerization produces low density composites). Unreacted monomer acts as plasticizer and weakens the matrix.

### 3.3. Raman Spectroscopy

**Figure 2:**
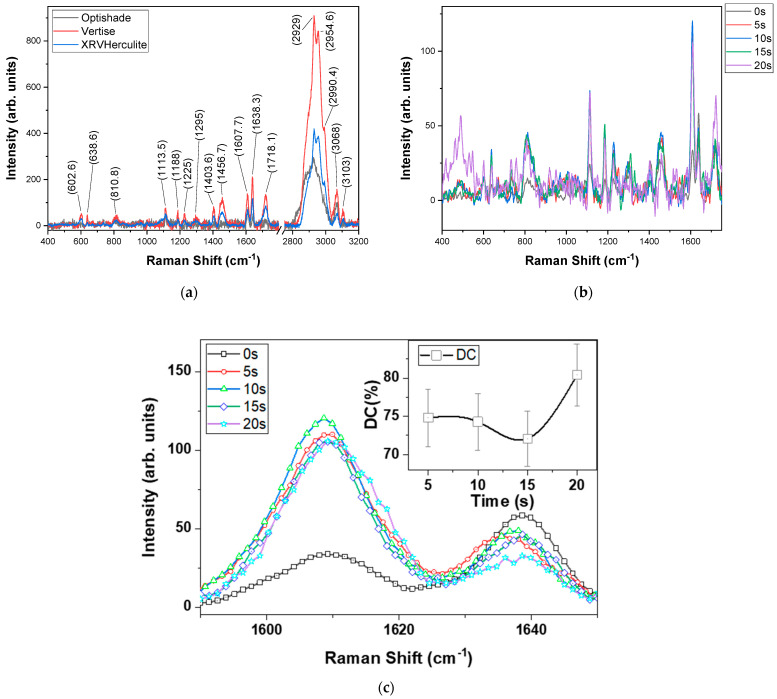
(**a**) Raman spectra at 532 nm for non-cured composites after luminescence correction; (**b**) Raman spectra for XRV Herculite samples during curing; (**c**) details of Raman spectra for XRV Herculite samples during curing showing 1608 and 1638 cm^−1^ bands. The inset shows the calculated degree of conversion.

The Raman spectra were recorded after each polymerization interval on the back of the sample (Figure 2) because it provided a smooth surface. The spectra of the VertiseFlow and Herculite were similar due to their close methacrylate concentration: VertiseFlow has a 30% methacrylate loading while Herculite has a 41% monomer loading. Optishade, however, showed different behavior: because of the 20% methacrylate loading of the composite, the spectrum displayed intense vibrational activity in the 600–3200 cm^−1^ range. A possible explanation was given by Willis et al. in 1969 [34]: the polymerized methacrylate molecule is both asymmetrical and amorphous, which lead to several conformational states.

To avoid any misleading data and bias from our part, we collected the Raman spectra of unpolymerized samples to identify the primary compositions of the three materials investigated here (Figure 2a). It can be clearly seen that the spectra have a similar shape for all the composites, showing the typical 638 cm^−1^ medium-intensity peak attributed to the C-COO in plane symmetrical deformation vibration, a 1113 cm^−1^ weak-intensity peak corresponding to the skeletal breathing mode (C-C-C), a 602 cm^−1^ peak attributed to the C-C-O symmetric vibration, a 810 cm^−1^ strongly polarized C-O-C bond, a 1456 cm^−1^ medium-intensity peak corresponding to the C-H vibrational mode in α-CH_3_, a 1403 cm^−1^ peak attributed to the CH_2_ bond, and a 1718 cm^−1^ peak attributed to the polarized C=O bond [34]. The assignment of the Raman bands is presented in Table 3.

To calculate the polymerization rate for the three composites, we needed to select two representative Raman bands: (1) one that corresponds to the polymerized composite (a peak that is attributed to a σ-bond characterized by the stretching or deformation mode of the polymer backbone → saturated chain) and a second band that corresponds to the un-polymerized composite (a peak attributed to a π-bond → un-saturated bond). Based on Figure 2c, we selected the 1608 cm^−1^ peak corresponding to the saturated C-C bond as “marker” of the cured composite [34] and the 1638 cm^−1^ band attributed to the un-saturated C=C bond [38]. By translating these peaks to the cured composites (e.g., Figure 2b and particularly 2c-Herculite), we observed that their ratio shifted during curing compared to the un-polymerized composite. This shift is given by the variables in the composition of the dental materials and the time of curing.

An important observation is that the uncured dental materials have a certain amount of polymerized material that act as polymerization sites and are responsible for the initialization of the curing (to kick-start the radical polymerization process) [39].

The polymerization rate was calculated based on Equation (2) [38], as follows:

(2) $$DC(degree\;of\;curing\;\%)=(1-\frac{R_{cured}}{R_{uncured}})\cdot 100$$

where the ratio of the cured/uncured composite is given as follows:

(3) $$R_{cured}=\frac{I_{{1638\;\mathrm{cm}}^{-1}}}{I_{{1608\;\mathrm{cm}}^{-1}}}$$

The results of the calculations are provided in Table 4, for each composite and for each curing time. Moreover, Table 4 also contains the methacrylate loading of each dental material.

**Figure 3:**
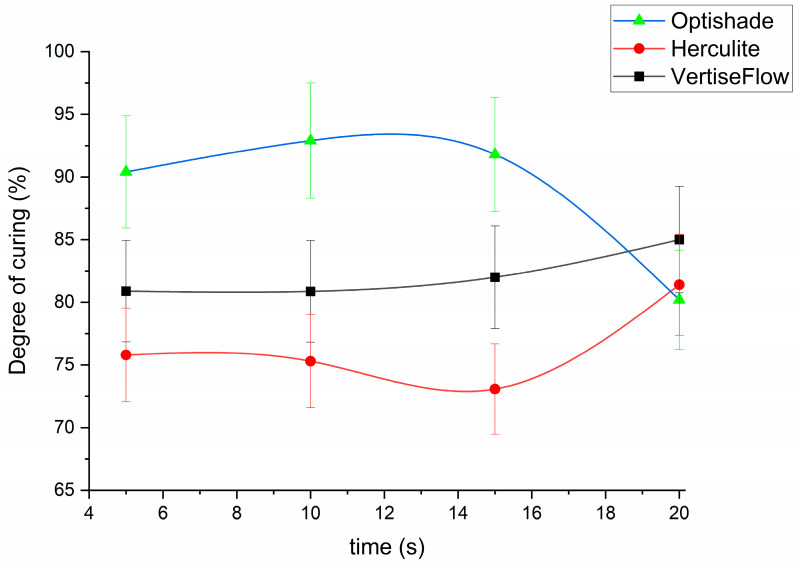
Variation of the degree of curing as a function of time for the three dental composites.

The highest DC value was calculated for OptiShade, which also indicated a stable polymerization process in time. At 20 s, the curing was slightly decreased because the reaction rate slowed due to shrinkage and breaking of the surface lattice (Figure 3) [40]. This high stability as a function of curing time is given by its composition of 80% nano-sized filler and 20% methacrylate monomer. Increasing the concentration of monomer to 41%, as it is the case of Herculite induces a curious behavior: the DC decreases from 74.8% (at 5 s curing time) to 72% (at 15 s) and increases sharply to 80.4% (at 20 s curing time). These results could be explained by the high content of inert mineral fillers, which interfere with the linear polymerization process of the resin [40,41]. By decreasing the amount of methacrylate in the dental material to 30% as it is the case for VertiseFlow, the DC value showed that the polymerization process was constant for the first 10 s, after which it increased slowly to 85%.

The amount of filler as well as the type of filler added to each product has a direct impact on the polymerization process. OptiShade has the highest amount of filler and exhibits the highest degree of curing, while VertiseFlow and Herculite show a lower polymerization grade. The difference comes from the size of the fillers: Herculite has a micro-range for its filler while OptiShade is composed of nano-sized fillers. Another difference that can explain the results obtained from the microhardness and density is the make-up of the filler: Herculite contains TiO_2_ and OptiShade contains only glass ceramics, while VertiseFlow contains small amounts of YbF_3_. Although fillers promote mechanical reinforcement and most importantly low polymerization shrinkage [42] for the dental composite, their performance is dependent on filler–polymer contact; if there are any kind of interfacial flaws, the stress point results in a decrease in flexural strength and fracture of the composite. In turn, as it ages and the fracture propagate in the bulk of the material, it produces breakdown of the restoration.

Comparing our data with the literature, we found that Taher reported similar DC values, in the range of 77–83% for Herculite, measured by Raman spectroscopy [43]. Unfortunately, the Optishade and VertiseFlow composites are relatively new and we could not find data for their DC values from Raman measurements. However, we found some reports for similar composites measured by FTIR with DC values in the 90% range [44]. We agree that are a lot of differences in DC data reported in the literature. The main sources of errors are related to differences in investigation method, sample preparation, Raman laser power or wavelength on sample, or even the post-curing polymerization effects [45].

Based on the Raman spectra, the optimal curing time for the composites are as follows: 20 s for Herculite and VertiseFlow, and 10 s for Optishade. By comparing these results with the ones from the density and microhardness measurements, one can observe that they have similar behaviour. Herculite has the highest density for all the materials investigated, coming close to that of human enamel after 20 s of curing, while VertiseFlow has the highest density after 15 s of curing time. OptiShade is the lightest material irrespective of the curing time (its density is lower than that of water). The microhardness measurements indicated that OptiShade has the best Vickers number after 15 s of curing, the same as VertiseFlow, while Herculite has the best microhardness number after just 10 s.

## 4. Conclusions

Three dental materials have been analyzed in terms of density, Vickers’ microhardness, and degree of curing by spectral monitoring via Raman spectroscopy at four different curing intervals. We attempted to rank them in order to establish their capacity to treat cavities in damaged teeth. Following the density measurements, we rated from best to worst, as follows: Herculite (20 s curing time) > VertiseFlow (15 s curing time) > Optishade. After the Vickers’ microhardness measurements, the rank became Optishade (15 s) ≥ VertiseFlow (15 s) > Herculite (10 s). The degree of curing indicated that Herculite (20 s) ≥ VertiseFlow (20 s) > Optishade (10 s). In conclusion, 10–15 s of curing time is optimal for Optishade to reach its best characteristics, while a curing time of 20 s is advised when using Herculite and VertiseFlow. Future studies will focus on the stability of the resin composites in different oral conditions (immersion of the polymerized resins in acidic/alcoholic synthetic plasma, or fracture induced by thermal shock).

## Figures and Tables

**Table 1:** Composition and release date of the dental composite materials used in this study.

Dental Materials	Composition	Filler Loading (wt%)	Release Date (On the Market)	Manufacturer
XRV Herculite^®^	Bis-GMA (bisphenol glycidil dimethacrylate) TEGDMA (triethylene glycol dimethacrylate) Prepolymerized filler (silica nanofiller-20–50 nm nanoparticles, barium submicron fillers-0.6 µm average size) Titanium Dioxide (TiO_2_) Organic pigments for shading	59%	October 2017	Kerr Corp., Orange, CA, USA
OptiShade^®^	BisGMA (bisphenol glycidil dimethacrylate) BisDMA (bisphenol A dimethacrylate) TEGDMA (triethylene glycol dimethacrylate) Filler (spherical silica and zirconia particles with effective particle size is 5–400 nm, 400 nm barium glass particles)	80%	June 2021	
VertiseFlow^®^	Bis-GMA (bisphenol glycidil dimethacrylate) GPDMA (glycerolphosphoric acid dimethacrylate) HEMA (hydroxyethyl methacrylate) Pre-polymerized filler (silanated barium glass, nano-sized colloidal SiO_2_, YF_3_)	70%	November 2021	

**Table 2:** Summary of the density measurements (n = 3) for the three dental materials at four curing intervals.

Time (s)	Ρ VertiseFlow (g/cm^3^) ± SD	Ρ Optishade (g/cm^3^) ±SD	Ρ Herculite (g/cm^3^) ±SD
5	1.0146 ± 0.05	0.4184 ± 0.02	0.5497 ± 0.02
10	0.8641 ± 0.04	0.733 ± 0.03	1.7716 ± 0.08
15	1.8606 ± 0.09	0.4527 ± 0.02	1.8408 ± 0.09
20	0.6367 ± 0.03	0.7442 ± 0.03	2.7015 ± 0.1

**Table 3:** Observed Raman bands and their assignments.

Raman Band	Assignment	Reference
602.6	ν_s_(C–C–O)	[34]
638.6	ν(C–COO)	[35]
810.8	νs(C–O–C)	[34]
1113.5	νa(C–O–C) and C–C skeleton backbone	[34,35]
1188	νa(C–O–C) and C–C skeleton backbone	[34,35]
1225	ν(C–O)	[34,36]
1295	ν(C–COO)	[34,36]
1403.6	CH_2_ twist or wag	[34]
1456.7	δa(C–H) of α–CH3δa(C–H) of O–CH3	[34,35]
1607.7	Aliphatic C-C	[37]
1638.3	Aromatic C=C	[37]
1718.1	ν (C=O)	[34]
2929	Combination band	[34]
2954.6	ν_s_ C–H stretching	[35]
2990.4	ν_a_ C–H stretching	[35]

**Table 4:** Calculation of the degree of curing for each dental composite at various curing times (5 s, 10 s, 15 s, and 20 s).

Composite Name	Monomer Composition	DC (% ± SD) (5 s)	DC (% ± SD) (10 s)	DC (% ± SD) (15 s)	DC (% ± SD) (20 s)
Vertise Flow	30%methacrylate	80.89 ± 4.04	80.87 ± 4.04	82 ± 4.1	85 ± 4.25
Herculite	41% methacrylate	74.8 ± 3.7	74.3 ± 3.7	72.08 ± 3.6	80.4 ± 4.0
Optishade	80% methacrylate	89.4 ± 4.47	91.9 ± 4.5	90.8 ± 4.54	79.2 ± 3.96

## Data Availability

Data are contained within the article.

## References

[1] Balhaddad A.A., Kansara A.A., Hidan D., Weir M.D., Xu H.H., Melo M.A.S. (2019). Toward dental caries: Exploring nanoparticle-based platforms and calcium phosphate compounds for dental restorative materials. Bioact. Mater..

[2] Silk H. (2014). Diseases of the mouth. Prim. Care Clin. Off. Pract..

[3] Pilcher L., Pahlke S., Urquhart O., O’Brien K., Dhar V., Fontana M., González-Cabezas C., Keels M.A., Mascarenhas A.K., Nascimento M.M. (2023). Direct materials for restoring caries lesions: Systematic review and meta-analysis-a report of the American Dental Association Council on Scientific Affairs. J. Am. Dent. Assoc..

[4] Wang L., D’Alpino P.P., Lopes L., Pereira J. (2003). Mechanical properties of dental restorative materials: Relative contribution of laboratory tests. J. Appl. Oral Sci..

[5] Chun K., Lee J. (2014). Comparative study of mechanical properties of dental restorative materials and dental hard tissues in compressive loads. J. Dent. Biomech..

[6] Chung S., Yap A., Koh W., Tsai K., Lim C. (2004). Measurement of Poisson’s ratio of dental composite restorative materials. Biomaterials.

[7] Jafarpour D., Ferooz R., Ferooz M., Bagheri R. (2022). Physical and Mechanical Properties of Bulk-Fill, Conventional, and Flowable Resin Composites Stored Dry and Wet. Int. J. Dent..

[8] Ramkumar Y., Hyoung L.H. (2022). Ranking and selection of dental restorative composite materials using FAHP-FTOPSIS technique: An application of multi criteria decision making technique. J. Mech. Behav. Biomed. Mater..

[9] Ramkumar Y., Mayank S., Anoj M., Seul-Yi L., Soo-Jin P. (2023). Selection and ranking of dental restorative composite materials using hybrid Entropy-VIKOR method: An application of MCDM technique. J. Mech. Behav. Biomed. Mater..

[10] Ramkumar Y., Anoj M. (2022). Comparative investigation of tribological behavior of hybrid dental restorative composite materials. Ceram. Int..

[11] Carvalho A., Pinto P., Madeira S., Silva F.S., Carvalho O., Gomes J.R. (2020). Tribological Characterization of Dental Restorative Materials. Biotribology.

[12] Maier E., Grottschreiber C., Knepper I., Opdam N., Petschelt A., Loomans B., Lohbauer U. (2022). Ulrich Evaluation of wear behavior of dental restorative materials against zirconia in vitro. Dent. Mater..

[13] Christian M., Soeren S., Klaus L., Matthias K. (2007). Wear of composite resin veneering materials and enamel in a chewing simulator. Dent. Mater..

[14] Paul L., Elke D., Kirsten V.L., Marleen P., Bart V.M. (2006). How to simulate wear?: Overview of existing methods. Dent. Mater..

[15] Heintze S.D., Zellweger G., Zappini G. (2007). The relationship between physical parameters and wear of dental composites. Wear.

[16] Yudaev P., Chuev V., Klyukin B., Kuskov A., Mezhuev Y., Chistyakov E. (2022). Polymeric Dental Nanomaterials: Antimicrobial Action. Polymers.

[17] Go H.B., Lee M.J., Seo J.Y., Byun S.Y., Kwon J.S. (2023). Mechanical properties and sustainable bacterial resistance effect of strontium-modified phosphate-based glass microfiller in dental composite resins. Sci. Rep..

[18] Yu H., Zhang C.Y., Cheng S.L., Cheng H. (2015). Effects of bleaching agents on dental restorative materials: A review of the literature and recommendation to dental practitioners and researchers. J. Dent. Sci..

[19] Rüdiger H., Björn T., Nina L., Bogna S. (2021). Impact of artificial aging by thermocycling on edge chipping resistance and Martens hardness of different dental CAD-CAM restorative materials. J. Prosthet. Dent..

[20] Gatin E., Luculescu C., Iordache S., Patrascu I. (2013). Morphological investigation by AFM of dental ceramics under thermal processing. J. Optoelectron. Adv. Mater.—JOAM.

[21] Sfeatcu R., Luculescu C., Ciobanu L., Balan A., Gatin E., Patrascu I. (2015). Dental Enamel Quality and Black Tooth Stain: A New Approach and Explanation by using Raman and AFM Techniques. Part. Sci. Technol..

[22] Gatin E., Ciucu C.G., Berlic C. (2008). Investigation and comparative survey of some dental restorative materials. Optoelectron. Adv. Mater.—Rapid Commun..

[23] Gatin E., Iordache S., Matei E., Luculescu C., Iordache A., Grigorescu C., Ilici R. (2022). Raman Spectroscopy as Spectral Tool for Assessing the Degree of Conversion after Curing of Two Resin-Based Materials Used in Restorative Dentistry. Diagnostics.

[24] Gatin E., Nagy P., Iordache S., Iordache A., Luculescu C. (2022). Raman Spectroscopy: In Vivo Application for Bone Evaluation in Oral Reconstructive (Regenerative) Surgery. Diagnostics.

[25] Gatin E., Nagy P., Paun I., Dubok O., Bucur V., Windisch P., Windisch P. (2019). Raman Spectroscopy: Application in Periodontal and Oral Regenerative Surgery for Bone Evaluation. IRBM.

[26] https://store.kerrdental.com/en-uk/.

[27] Jafarzadeh T.-S., Erfan M., Behroozibakhsh M., Fatemi M., Masaeli R., Rezaei Y., Bagheri H., Erfanz Y. (2015). Evaluation of Polymerization Efficacy in Composite Resins via FT-IR Spectroscopy and Vickers Microhardness Test. J. Dent. Res. Dent. Clin. Dent. Prospect..

[28] Maas M., Alania Y., Natale L., Rodrigues M., Watts D., Braga R. (2017). Trends in restorative composites research: What is in the future?. Braz. Oral Res..

[29] Vaizoglu G., Ulusoy N., Alagöz L. (2023). Effect of Coffee and Polishing Systems on the Color Change of a Conventional Resin Composite Repaired by Universal Resin Composites: An In Vitro Study. Materials.

[30] Weidmann S.M., Weatherell J.A., Hamm S.M. (1967). Variations of enamel density in sections of human teeth. Arch. Oral Biol..

[31] Nicholson J.W. (2023). Ytterbium (III) Fluoride in Dental Materials. Inorganics.

[32] Dias A., Gonçalves C., Caço A., Santos L., Piñeiro M., Vega L.F., Coutinho J.A., Marrucho I.M. (2005). Densities and Vapor Pressures of Highly Fluorinated Compounds. J. Chem. Eng. Data.

[33] Popov L. (2010). Physical Principles and Applications.

[34] Willis H.A., Zichy V.J.I., Hendra P. (1969). The Laser-Raman and Infra-red Spectra of Poly(Methyl Methacrylate). Polymer.

[35] Chaurasia S., Rao U., Mishra A.K., Sijoy C.D., Mishra V. (2020). Raman spectroscopy of poly (methyl methacrylate) under laser shock and static compression. J. Raman Spectrosc..

[36] Xu X.S., Ming H., Zhang Q.J., Zhang Y.S. (2002). Properties of Raman spectra and laser-induced birefringence in polymethylmethacrylate optical fibres. J. Opt. A Pure Appl. Opt..

[37] Shin W.S., Li X.F., Schwartz B., Wunder S.L., Baran G.R. (1993). Determination of the degree of cure of dental resins using Raman and FT-Raman spectroscopy. Dent. Mater..

[38] Miletic V., Santini A. (2012). Micro-Raman spectroscopic analysis of the degree of conversion of composite resins containing different initiators cured by polywave or monowave LED units. J. Dent..

[39] Damoun S., Papin R., Ripault G., Rousseau M., Rabadeux J.C., Durand D. (1992). Radical polymerization of methyl methacrylate in solution monitored and studied by Raman spectroscopy. J. Raman Spectrosc..

[40] Panpisut P., Liaqat S., Zacharaki E., Xia W., Petridis H., Young A.M. (2016). Dental Composites with Calcium / Strontium Phosphates and Polylysine. PLoS ONE.

[41] Xia W., Razi M.M., Ashley P., Abou Neel E.A., Hofmann M.P., Young A.M. (2014). Quantifying effects of interactions between polyacrylic acid and chlorhexidine in dicalcium phosphate—Forming cements. J. Mater. Chem. B.

[42] Elfakhri F., Alkahtani R., Li C., Khaliq J. (2022). Influence of filler characteristics on the performance of dental composites: A comprehensive review. Ceram. Int..

[43] Taher R., Moharam L., Amin A., Zaazou M.H., El-Askary F.S., Ibrahim M.N. (2021). The effect of radiation exposure and storage time on the degree of conversion and flexural strength of different resin composites. Bull. Natl. Res. Cent..

[44] Rezaei S., Abbasi M., Mahounak F.S., Moradi Z. (2019). Curing Depth and Degree of Conversion of Five Bulk-Fill Composite Resins Compared to a Conventional Composite. Open Dent. J..

[45] Par M., Gamulin O., Marovic D., Klaric E., Tarle Z. (2015). Raman spectroscopic assessment of degree of conversion of bulk-fill resin composites—Changes at 24 hours post cure. Open Dent..

